# An Evidence-Based AI Virtual Assistant for Young People With Attention Deficit Hyperactivity Disorder: Co-Design and Prototype Development

**DOI:** 10.2196/85013

**Published:** 2026-09-10

**Authors:** Eleanor F Bryant, David Hallett, Emily Nielsen, Tali Evans, Jacqueline Rees-Lee, Nicole Riley, Tamsin Newlove-Delgado, Anna Price

**Affiliations:** 1 University of Exeter Medical School University of Exeter Exeter United Kingdom; 2 Digital Futures Torbay and South Devon NHS Foundation Trust Devon United Kingdom; 3 School of Engineering Mathematics and Technology University of Bristol Bristol United Kingdom; 4 SmartADHD Research Advisory Group University of Exeter Devon United Kingdom

**Keywords:** attention deficit hyperactivity disorder, neurodevelopmental disorders, artificial intelligence, health services accessibility, health equity, interdisciplinary research, patient participation, innovation, underserved population, generative AI

## Abstract

**Background:**

Though attention deficit hyperactivity disorder (ADHD) is thought to be the most prevalent neurodevelopmental disorder in young people worldwide, there are inequalities in access to psychoeducation and health care support. One way to improve access, potentially increase engagement, reduce health care inequalities, and enhance care is by co-developing digital responsive interventions. These have the potential to support long-term condition management and to act as an adjunct to usual care. Virtual assistants that use large language models can provide information in response to questions and learn to tailor communication to suit an individual user’s needs. This can be especially valuable for people with ADHD who often struggle to regulate attention and can experience communication challenges. Involving people with lived experience in the co-design process is crucial for the development of effective digital interventions. Therefore, this article explores the views and preferences of young people with ADHD and their supporters from the United Kingdom who collaborated with researchers to co-design a prototype chatbot.

**Objective:**

This study aimed to co-develop an evidence-based chatbot prototype, intended to help young people with ADHD thrive through improved access to health care information, psychoeducation, and self-management strategies.

**Methods:**

An interdisciplinary team was established, including researchers, software developers, clinicians, and lived experience collaborators. Research advisory and working groups were set up in ways that facilitated flexible involvement. Following the person-based approach, guiding principles were established, and workshops were held with young people with ADHD and supporters of young people with ADHD to co-develop an early prototype. Feedback was sought via think-aloud interviews with lived experience collaborators.

**Results:**

In total, 9 experts by lived experience and 3 health care professionals chose to engage in workshops, and this feedback informed the development of a SmartADHD chatbot prototype. An off-the-shelf chatbot (GPT-4o hosted on Convai) was trained using resources from the National Health Service (NHS). Overall, 6 experts by lived experience engaged with think-aloud interviews, providing feedback on the prototype conversational flow and feel, the avatar, the text-to-speech, the chatbox feature, and the content of the messages. Seven recommendations are made for future development, which will inform the SmartADHD program of work.

**Conclusions:**

These findings provide rich data on the preferences of people with ADHD. Specific recommendations for a chatbot for young adults with ADHD have not been investigated before with young people, making this study a novel contribution to the field. These findings provide an excellent foundation for chatbot development for this group and may be relevant for those developing digital tools for people with ADHD across the lifespan and other neurodevelopmental conditions. Further work is required to elucidate the views of health care professionals and identify the limits of the technology before subsequent evaluation.

## Introduction

### ADHD Prevalence and Outcomes

Attention deficit hyperactivity disorder (ADHD) is the most common neurodevelopmental disorder in England and affects nearly 7% of adults globally [1]. It is characterized by impairment across inattention, impulsivity, and hyperactivity. Characterizations of ADHD suggest there are three subtypes—inattentive, hyperactive, and combined subtype, with heterogeneous traits across and within individuals [2].

young people with ADHD are likely to face poorer outcomes in life functioning across domains including academia, antisocial behavior, driving, nonmedicinal drug use or addictive behavior, obesity, occupation, service use, self-esteem, and social functioning outcomes. When compared to people without ADHD, those with ADHD face poorer outcomes whether their condition is treated or untreated [3].

ADHD was previously thought to be a disorder limited to childhood, but more recent understanding suggests that in around 3% of the population, ADHD with a childhood onset is maintained into adulthood, equating to nearly 140 million people worldwide. When symptoms or traits are considered, regardless of childhood diagnosis, this number could include an additional 366 million affected adults [4].

There is ongoing discourse regarding the potential overdiagnosis of ADHD in the United Kingdom; however, evidence suggests that, though a small number of cases may incorrectly be attributed to ADHD, diagnosis rates remain “substantially below the population prevalence in the United Kingdom, providing no evidence at present that ADHD is overdiagnosed at a population level” [5].

### ADHD Care Provision in the United Kingdom and the Role of Digital Health

ADHD health care provision is failing patients [6]. This issue is exacerbated for people with ADHD in transition between childhood and adulthood (ie, aged 18-24 years) [7].

There are long waiting lists for ADHD diagnosis, with minimal support for those on the waiting list [8]. Not only do young people have better outcomes when they receive treatment, but being on a long waiting list can have negative outcomes. These individuals are more likely to use more health care resources than others [9], and being on a waiting list can cause financial stress due to loss of employment, social isolation, lack of support, and reduced quality of life [10].

Being on the waiting list has personally caused me to have serious mental health problems because I feel like I’m not getting the help I need to carry on through life. [A girl aged 16 years]

8

Even after diagnosis, pharmacological and psychosocial support can be lacking and is often highly variable depending on the services available in the area where the young person lives [11].

Systemic barriers that face neurodivergent young people when accessing the health care system further decrease the likelihood of accessing support [12]. Compounding this, ADHD is highly heritable, and it is estimated that 44% of people with ADHD also have at least one parent with ADHD, meaning that barriers persist for young people who need parental support to access the health care system [13].

There is potential for carefully curated bespoke digital health technologies to help address unmet need for young people with ADHD and reduce gaps in service provision. Digital technologies represent a financially viable solution, with some methods having demonstrated cost-effectiveness [14]. They also offer the potential to help support young people with ADHD but lack a rigorous evidence base [15]. Mobile phone apps have been shown to create behavior change and engagement by harnessing behavior change techniques [16,17]. Other digital health interventions (DHIs) have been effective for ADHD [18]. Furthermore, the National Health Service (NHS) is encouraging increasing digitalization as an adjunct to usual care, with the July 2025 “Fit for the Future” report recommending a shift from “analogue to digital” care [19].

Nevertheless, current digital information can be unreliable, untrustworthy, and not evidence-based, and yet consumed frequently by young people with ADHD [20-23]. Therefore, there is a need for ADHD-specific DHIs that are evidence-based.

Large language models (LLMs) are increasingly being used in DHIs and have been used for a variety of purposes within mental health support [24,25]. They have shown promise to support young people with ADHD. Chatbots can break down tasks into manageable chunks, reducing overwhelm [26,27]. They can offer on-demand and consistent support [28,29], emotionally neutral feedback that avoids judgment [30,31], and engagement and motivation [32,33]. Users often find chatbots more fun and less intimidating than traditional tools [29,34]. There is also the option for personalization in terms of the user’s pace, language, and preferences [35-37], as well as cultural, linguistic, and neurodiverse needs [35,36]. All the above are features that could make chatbot interventions appropriate for people with ADHD, who may face challenges with being easily distracted, forgetful, and finishing tasks.

Therefore, we suggest that an evidence-based chatbot could improve communication of health care information for individuals with ADHD who may have differing needs. A well-designed chatbot could be accessible to people from different backgrounds and provide a trustworthy alternative to the high volumes of digital misinformation and unreliable resources that exist online [20]. Chatbots have previously been used to provide support for mental health conditions (these are advanced in the eating disorder literature especially) [38], but this work is novel in being the first that the authors know of that aims to act as an adjunct to ADHD care, and particularly with respect to young people in this transitional period between services.

However, there are important concerns around safety after problems have arisen with other chatbots. There are examples of generative AI (GenAI) tools enabling users to generate harmful eating disorder content [39]. Indeed, a chatbot launched in 2023 by the National Eating Disorder Association in the United States was taken down shortly after launch as it provided harmful advice to users about losing weight and dieting [40]. This tool was developed by academics, who claim that the safeguards they set up should not have allowed this to happen, and that edits had been made to the chatbot function before publication. Further demonstrating that chatbots for mental health are in their infancy, this was a rule-based chatbot, which, unlike GenAI models, was developed for precision. Rule-based AI is transparent and inflexible—it always gives the same answer to the same question. This shows that significant improvement is required before rule-based chatbots, let alone GenAI, are safe for use as patient-facing medical devices.

Preliminary evidence suggests that the feasibility and acceptability of DHIs for young people with ADHD are strongest when co-developed with experts with lived experience (LE) [41]. This underlines the need for a development, design, and implementation process that uses co-development methods. We will outline the processes that we followed in a linked paper. Despite rapidly evolving challenges and solutions in relation to AI-based health care, it remains essential that early chatbot development work involves people with LE, digital experts, and health care professionals (HCPs). Co-production is focused on increasing understanding about the priorities, needs and preferences of young people with ADHD for a chatbot that will help them manage their condition safely, to access appropriate health care, and to thrive.

### Improving Access to Care and Using Digital Tools to Enhance Service Provision

Consultation with young people has shown that they favor digital apps as a source of health care information, and that this should contain psychoeducation (information about the condition) and information about accessing health care services in the United Kingdom [42,43].

Health care providers also favor an online solution, as this allows information to be kept up-to-date more easily and the ability to signpost to one trusted source [11,44]. Clinicians also wish to improve the sharing of information with young people [45], and support digital interventions as an adjunct to primary care if “reliable and well-curated” [44]. Digital resources can be updated frequently when new information comes to light, can be accessed quickly by the patient, and can give an LE perspective that most general practitioners (GPs) cannot. Digital interventions could also reduce administrative tasks for consultants, as common queries and requests for information could be fielded through these interventions.

The provision of information about how to access health care is recommended by the National Institute for Health and Care Excellence (NICE) and the NHS; nevertheless, accessing the health care system in the United Kingdom, whether public or private, can be confusing for young people and supporters. Supplying a web portal for community-based ADHD care saw parents rate improved ADHD symptoms and improved ADHD care quality, showing that digital interventions can improve access to health care [46]. The chatbot may fill this gap by improving communication through the synthesis of information and the ability to tailor communication. This has the potential to be prescribed through a social prescribing model for health care access equity.

### Aims of This Co-Production Work

This paper describes early co-production work, conducted between January and September 2025, and reports on the preferences of young people with ADHD and their supporters. Preferences relate to an AI chatbot that is trained on evidence-based materials specifically to give psychoeducational, behavioral, and health care information for young people aged 16-25 years with ADHD. The information reported in this article reflects conversations with collaborators with LE of ADHD and reflects proof of concept only; this is development work, and extensive future research would be required to ensure the chatbot was safe and fit for purpose.

## Methods

### Interdisciplinary Team Development

An interdisciplinary team was established as part of the broader SmartADHD project [47]. This included project leads from the University of Exeter and the Digital Futures Lab, an LE expert, and a human-computer interaction (HCI) researcher from the University of Bristol. LE and HCP research advisory groups (RAGs) were set up in line with UK Standards for Public Involvement, with linked working groups (WGs) whose members could contribute flexibly to research activities [48]. For details, see terms of reference and role descriptors (see Multimedia Appendix 1 and Multimedia Appendix 2 for examples). A mission statement was co-developed, and training resources (eg, on the basic design principles of HCI) were provided for researchers and lived-experience colleagues and shared via YouTube and the study website [49]. Meetings were held to discuss research, software engineering, and ADHD specific processes and experiences, to help build a shared language across disciplines and experience and, through an iterative process, produce a combined direction of travel and map a perceived ideal outcome.

### Approach

The wider research studies with which this co-design exercise is associated adopt the meta-paradigm of critical realism. This states that an objective reality exists independently of our knowledge of it, and that while there are real and actual truths about the nature of the world, it is impossible to completely understand this reality [50]. Critical realism is an appropriate and pragmatic approach commonly used in applied health care research and supports the need for theory-driven, person-focused, and context-aware intervention development [51].

With this in mind, the person-based approach (PBA) was used to guide digital intervention development, as this is an established method for combining evidence, theory, and person-based research [52]. As part of a larger body of SmartADHD work, a review and synthesis of relevant literature was conducted by the research team (AP, TE, and EB), and a summary was created of relevant primary and systematic review evidence. A theory of change model was drafted, based on the behavior change wheel and intervention planning table populated, covering target behaviors, for uptake, engagement, and knowledge use (Multimedia Appendices 3 and 4) [53]. Guiding principles for intervention design (covering user characteristics, design objectives, and key features) were drafted and collaboratively reviewed over multiple iterations. Gaps in knowledge were identified in relation to user needs (EN) and noted for discussion during workshops. Details of PBA documents will be provided in a linked SmartADHD publication.

### Working Group

A diverse LE working group of 13 people was formed to co-develop the Chatbot prototype, with work supported and guided by SmartADHD RAG members and the wider study team. The working group, which included 9 young people with ADHD and 4 supporters (parents), was made up of a mixture of individuals with previous involvement in the Science of ADHD and Neurodevelopment (SAND) collaboration, including members of the “Mapping ADHD services in primary Care” (MAP) study RAG, and new members [54]. Members were identified via previous involvement, study networks, and word of mouth, and invited to express interest in involvement via a Microsoft Form sent as a link [55]. Members were provided with terms of reference, links to SmartADHD resources, an introductory meeting, and a schedule of planned meetings. We engaged with colleagues through a range of different methods to help keep all team members well-informed, including frequent reminders. Meeting invites were sent via email, Microsoft Teams, WhatsApp (Meta), and text, depending on member preference [56]. Attendance and demographic information for these groups can be found in Tables 1 and 2. Some members attended several meetings; some did not attend any.

**Table 1:** Demographics of the lived experience working group from which meeting attendees were drawn.

Categories^a^ and subcategories			Attendees, n
**Experience**			
	Has ADHD^b^	9	
	Supports someone with ADHD	4	
**Age (years)**			
	18-24	4	
	25-34	3	
	35-44	0	
	45-54	3	
	55-64	1	
	65 or older	0	
	Unknown	2	
**Gender**			
	Man	4	
	Woman	9	
	Other	0	
**Ethnicity**			
	White British	8	
	Mixed or multiple ethnic groups	3	
	Black, Black British, Caribbean or African	1	
	Asian or Asian British	1	
**Region**			
	North East	0	
	North West	2	
	Yorkshire and the Humber	0	
	East Midlands	2	
	West Midlands	1	
	East of England	1	
	London	0	
	South East	1	
	South West	5	
	South East Wales	1	

^a^Demographic information was self-identified by collaborators in response to demographic questions. We have reported the demographic information of all members of the working groups to avoid identifying individual members, though not all members attended group meetings or workshops.

^b^ADHD: attention deficit hyperactivity disorder.

**Table 2:** List of workshop attendees.

Workshop name	Attendees
Identity and guiding principles	Lived experience research advisory group facilitator Expert by lived experience; supporter Expert by lived experience; young person Expert by lived experience; young person Health care professional; GP^a^ Health care professional; senior specialist ND^b^ coach, mentor, trainer, and facilitator Health care professional; ADHD^c^ nurse specialist and ADHD service manager GP partner
Tailoring the chatbot to underserved groups	Expert by lived experience; young person Expert by lived experience; young person
Detailed content workshop	Lived experience research advisory group facilitator Expert by lived experience; supporter Expert by lived experience; young person Expert by lived experience; young person Working group member; supporter of children with ADHD; has ADHD
The identity of the chatbot	Working group member; supporter of child with ADHD Expert by lived experience; young person Working group member; supporter of children with ADHD; has ADHD
Health care professionals consultation	Health care professional; senior specialist ND coach, mentor, trainer, and facilitator Researcher and health care professional Health care professional; ADHD nurse specialist and ADHD service manager

^a^GP: general practitioner.

^b^ND: neurodiverse.

^c^ADHD: attention deficit hyperactivity disorder.

### Workshops

Five workshops were held with LE collaborators on chatbot co-development, covering guiding principles, tailoring for underserved groups, language and content, and chatbot identity. A further workshop was held with HCP collaborators. These are built on the guiding principles and theory of change documents from the PBA. Sessions were run flexibly via Microsoft Teams, often including presentation slides, with notes made on the slides by the meeting facilitator. Minutes were made and shared with attendees afterward. Meetings were recorded to add detail to notes after each workshop. Feedback from the workshops was summarized and translated by the research team, following content analysis methods, into a specification document to inform prototype development. The workshops and the resulting feedback are outlined in Table 3.

**Table 3:** A table of workshops that were carried out with lived experience collaborators, the topics that were discussed in this workshop, and the feedback that resulted in specifications for the prototype chatbot.

Workshop name	Topics discussed	Changes made
Identity and guiding principles	Name of the project and app Logo of the project and app Guiding principles	After the workshop, SmartADHD^a^ was chosen as the name with agreement from collaborators. After the workshop, a logo was designed by a young person in the research advisory group, finessed by the team, and adopted Guiding principles confirmed
Tailoring the chatbot to underserved groups	User personas Potential and limitations of the chatbot for different users How this relates to underserved groups	None made Chatbot should summarize and chunk complex information. Chatbot should personalize content dynamically based on interaction history and user profile. Options for low literacy or tech literacy users
Detailed content workshop	Language and dealing with difficult or sensitive topics. Looking at an example app and giving feedback on tone, word choice, level of detail	Unambiguous, plain language will be used throughout the app Technical terms explained before use (eg, “increasing medication dose [titration]”) Use validating language, avoid language such as “deficit”, “low-functioning” or “disorder” outside clinical definitions
The identity of the chatbot	Mock-ups made based on feedback from the previous chatbot meeting The communication identity of the chatbot The visual identity of the chatbot	None made. Capable of being “fun” if context-appropriate and done well Avoid emojis; maintain a professional yet engaging tone This will be different by individual. Emphasis should be on ability to customize
Health care professionals consultation	Consider how to make the chatbot accessible to young people who have had negative experiences with services	Consider how the chatbot will advise those in the youth justice system, those not registered with a GP or those with additional mental health problems

^a^ADHD: attention deficit hyperactivity disorder.

### Iterative Prototype Development

Following the workshops, a technical specification was developed to guide chatbot initial development, and then refinement, with consideration of 1. content from workshops, 2. information collected from the person-based approach, and 3. Basic Design Principles of HCI. Discussions were held with the software engineer (DH) and wider team, prioritizing feature development and considering technical and resource constraints. A final prototype was produced and trialed by research and HCI colleagues (EB and EN). Technical information about the chatbot can be seen in Textbox 1.

**Chatbot technical information.**

An existing online service was leveraged to develop the proof of concept. Although it was within the developers’ capability to run a local model and make use of text to speech and speech to text systems, the use of an online package significantly increased the functionality available, particularly in terms of knowledge banks and reliable character control. It is important to note that the development of a bespoke large language model (LLM) trained specifically for this purpose would require investment in the order of tens of millions of pounds and was neither practical nor necessary for this application. Instead, the team leveraged a pretrained foundation model with a structured prompting and knowledge retrieval approach.

This method of using pretrained off-the-shelf LLMs with prompting is a fairly standard method of operating used by many clinical AI-based systems including several which have been approved as medical devices. The chatbot was based on GPT-4o using the Convai [57] platform, which provides memory and integration layers around a selected LLM. Convai acts as a middleware platform, providing session memory, character definition, knowledge retrieval, and input/output integration layers around a selected foundation LLM.

DH defined a character mind layer including personality, goals and backstory, behavioral traits, and access to knowledge banks. The core description can be seen in Multimedia Appendix 5. A knowledge bank was used with the aim of mitigating hallucination risk and providing further expertise beyond the training data; The data in the knowledge bank is passed to the LLM as context alongside the message from the user and informs processing and response generation. Documents uploaded to the knowledge bank are retrieved and injected into the LLM prompt as contextual information alongside the user's message, a technique commonly referred to as retrieval-augmented generation (RAG). In this case, National Institute for Health and Care Excellence (NICE) and National Health Services (NHS) documents and website were uploaded to the platform (Multimedia Appendix 6).The chatbot was limited in the advice it could give and based its answers on the trusted sources provided.

The Convai system allows user input through speech to text or typed messages. Responses are then provided either as plain text, via speech to text, or driving a lip-synced animated avatar. A number of avatar presets are available within the platform, including varying professions, backgrounds, and costumes. Limited by the platform and bearing in mind the eventual purpose of the chatbot (to provide trusted medical information to young people from underserved groups), the avatar chosen was a female with a dark skin tone wearing blue medical scrubs (Multimedia Appendix 7).

### Think-Aloud Interviews

Following established PBA methods, think-aloud interviews were carried out with LE collaborators to user-test the first prototype of the chatbot and gather feedback on user experience [58]. A set of feedback prompts (see Feedback Points in Multimedia Appendix 8) was developed by EB, trialed with an HCI expert (EN) in a pilot interview, and reviewed by the team. Then, working group members were invited by email to book a 1-hour online meeting with researcher EB, with a link to a Microsoft bookings page. Reminders were sent via email or WhatsApp, in line with individual preferences. EB is a female research assistant who holds a BSc and had over 3 years’ experience in mental health research at the time of interviews. EB had an existing relationship with all members of the working group as she was involved in coordinating the group. Collaborators had been involved in conversations around the chatbot from the conceptualization of the project, some more directly than others. Collaborators knew about EB’s background and role on the project as the coordinator of the public involvement, research, and digital teams. For the purpose of this initial user testing, users were made aware that the chatbot was a prototype. They were only given access to this during the session and used it in the presence of a researcher. The chat history was saved within a password-protected login, which could only be accessed by the developers and research team.

Six think-aloud interviews were conducted over Microsoft Teams, with 3 young people with ADHD (collaborators 2, 3, and 6) and 3 supporters (parents) of people with ADHD (collaborators 1, 4, and 5). Three collaborators were from South West England, with the remaining collaborators from the North West, North East, and East of England. Four were female, and two were male. Collaborators brought a diversity of experience in relation to previous familiarity with Chatbots and with this project. One supporter had not used a Chatbot before, while the rest were experienced users. Some attendees were active members of the working group and had been involved in prototype development, while 2 members - though involved in the SmartADHD project (either in the WG or RAG) - had not participated in chatbot-specific workshops, so the chatbot was completely new to them. Though they may have seen a screenshot of the chatbot previously if involved in workshops, no collaborators had directly interacted with the SmartADHD chatbot before.

No repeat interviews were carried out. Informal field notes were made by EB throughout interviews. The think-aloud interviews were recorded with permission from collaborators and transcribed automatically; these were not sent to collaborators for comment as it was not essential for the transcripts to be completely accurate, and recordings could be consulted when the researchers might have been unsure. We (EN, EB, and AP) adopted a deductive analysis approach to categorize feedback, to provide specific information to inform future design iterations. In particular, EB deductively identified feedback using Lumivero’s NVivo 14 [59], in relation to features that worked well, features that did not work well, future developments, and suggestions on every segment of text. These were categorized into the following predefined topics relating to components of the chatbot:

1. The overall conversation
2. The avatar
3. The speech
4. The content
5. The chat box (text input)

### Ethical Considerations

This study describes patient and public involvement and engagement (PPIE) co-production research to inform early co-development of a chatbot prototype for people with ADHD. Public involvement is defined by The National Institute for Health and Care Research (NIHR) as research being carried out “with” or “by” members of the public, and public engagement is described as the “myriad ways in which the activity and benefits of…research can be shared with the public” [60]. In the United Kingdom, “ethical approval is not needed for PPIE activities”, which are considered essential as they facilitate more impactful research outcomes [60,61]. Further clarification can be found in guidance provided by the co-production collective:

“Existing research ethics processes and policies focus on the relationship between researcher and participant and are not concerned with the consideration of ethical issues in public engagement and Patient and Public Involvement and Engagement” [62].

In the methods described above, team members were consulted as expert and equal contributors to the design process and provided iterative design feedback via think-aloud interviews, rather than participating as research subjects and providing data. This work meets UK Health Research Authority best practice principles for public involvement by engaging experts with LE in early stages of intervention co-development [63], and laying strong foundations for jointly defining the ‘rationale, scope, design, and conduct’ of future planned research to develop and evaluate a Chatbot for young people with ADHD.

This PPIE and co-production research was conducted in line with international ethical guidelines [48]. It followed UK Research and Innovation key principles for co-production in research [64], and the established framework of UK Standards for Public Involvement in Research [48]. This included, for example, working together, using clear communication, providing inclusive opportunities, establishing equitable partnerships, and supporting learning.

LE colleagues formed a key part of the interdisciplinary research team and contributed as equal partners. As described above, terms of reference and role descriptors were provided for all RAG and WG members. LE colleagues received payment in recognition of their time, in the form of online shopping vouchers, while HCPs were provided with certificates of continuing professional development. Collaborators were provided with opportunities to learn more about research, receive feedback on the impact of their involvement, and were supported to contribute to outputs, including this publication.

## Results

### Results: Impact of the Views of Experts by LE

Findings from the 5 workshops, which brought together members of the working groups to co-design the chatbot, are summarized in Table 3 (see above), with details provided in the Feedback From Initial Co-Design Workshops section. Feedback from think-aloud meetings with 6 collaborators, where they were invited to trial the co-developed early chatbot prototype is provided in the Think-Aloud Interviews—Feedback on the Initial Prototype section.

#### Feedback From Initial Co-Design Workshops

From across the 5 workshops, we (EB, TE, and AP) drew together feedback about the chatbot that we then communicated with the tech developer (DH) as a product specification. These covered the chatbot as a whole, its purpose, design, content, and any features that would facilitate integration with a future SmartADHD app. The specifications document can be seen in Multimedia Appendix 9.

#### Whole Chatbot

Feedback that was relevant to the whole chatbot included the focus or emphasis, the personality or tone, the ability for it to function as a stand-alone technology, and to serve underserved groups.

Collaborators felt that the emphasis of the chatbot should not be the new AI technology, but the ability to help or offer aid to people with ADHD. Collaborators preferred the chatbot to have a friendly and warm personality but maintain professionalism. It could use humor, but only if this was done appropriately and within the limits of the technology. Collaborators wanted the chatbot to be able to act as a stand-alone device for use either with or without the associated app. Collaborators felt that the chatbot should be able to understand contextual factors that may be relevant to the ability of people from underserved groups to access relevant information from the chatbot. For example, cultural factors such as religion and attitudes to ADHD, or co-occurring health conditions.

#### Chatbot Purpose

Collaborators’ views on the purpose of the chatbot included the inclusion of behavioral support interventions, health care access information, using evidence-based, credible, and trustworthy sources, less well-known information, and the ability of the chatbot to summarize this information and tailor to the user’s needs.

Collaborators were of the view that the chatbot should be able to provide behavioral advice and strategies to users, broken into step-by-step instructions. Collaborators felt that the chatbot should be able to provide information about accessing appropriate health care in the UK. For example, seeking a diagnosis, or following up with a GP. The chatbot should not provide medical advice, but signpost to evidence-based sources and suggestions of how to navigate the system. Collaborators thought that the chatbot should not repeat information that they deemed ‘basic’, and that it should help reveal information that they might not have been able to access previously. A key purpose of the chatbot should be to condense, chunk, or summarize information, making it shorter and faster to read. The chatbot should not give one-size-fits-all information and should be able to tailor information to the user. Collaborators had different views about how curious the chatbot should be initially or, conversely, should be able to amend previous answers once it is asked to apply further information to these. The chatbot should not only be able to tailor the information given to the user, but also the way this is communicated, for example in the formality or complexity of the language.

#### Customization

Customization was a topic which came up in nearly every workshop in one way or another. Users wanted to be able to customize the appearance of the avatar, the level of detail or specificity of information given to them, to discard or encourage information or types of communication, to allow the chatbot to personalize responses based on current life events and phases, and the same for any co-occurring conditions. Users emphasized that all people with ADHD are different, and that no one thing would work for all users; therefore, customization would be paramount.

Collaborators thought that if the user was clicking on lots of hyperlinks, more detailed information should be provided in the future. They also thought that users should be able to rate messages with a thumbs-up or thumbs-down to indicate whether they are helpful. Thus, this would inform future chatbot response tailoring. If the user reveals information about themselves, for example, that they are about to start University, the chatbot should be able to give information tailored to that specific phase of life in the future, when users ask for advice. There should be the ability to customize settings, for example, use a red and green screen filter for color blindness.

#### App Integration Features

For future integration with an app, collaborators suggested that streaks or reminders and notifications to check the chatbot could be useful for improving engagement but could also create feelings of pressure and guilt in users. Notifications that were too frequent could be annoying.

### Think-Aloud Interviews—Feedback on the Initial Prototype

Feedback was collated from the transcripts and notes of think-aloud meetings with 6 collaborators, which captured their real-time experiences and views as they interacted with the early chatbot prototype. Users provided rich feedback in relation to strengths, weaknesses and opportunities for development.

From these think-aloud meetings, five topics were identified by EB and TE: the chat box and typing interface, the text-to-speech function, the conversational flow and feel, the avatar, and the message content. Users were generally very positive about the content of the chatbot and liked the design of the interface (aside from some technical problems) and the text-to-speech, but there was some specific feedback on how the voice could be improved. There were mixed views on the avatar. The flow of the conversation was reported as being generally good, but users did not like it when the chatbot session “crashed” and erased the chat history. Crucially, though they liked the content of the messages, collaborators were unable to identify where the information had come from when asked. A summary of the feedback from the think-aloud interviews, with illustrative quotes, can be found in Table 4, with further details provided below. A screenshot of the chatbot interface can be seen in Multimedia Appendix 7.

**Table 4:** Summary of think-aloud engagement interview findings.

Topics, feedback, and illustrative quotes				Collaborators, n
**Conversational flow and feel**				
	**Strengths were that the messages were deemed appropriate lengths for the information**			
		Yeah, they were like a little bit lengthy, but I was asking quite lengthy questions, and it was trying to give me technical information. So, I think it makes sense.	3	
		“OK, so it did a really nice job here because I asked two separate questions, but actually it made more sense for them to be put together and it put them together for the response.”	1	
	**Weaknesses were mainly to do with technical difficulties, including the chat restarting**			
		“I think that was the only thing that I didn't like that [it] kept on restarting and there's nothing that you could carry on from.”	2	
	**Opportunities for development included improved use of memory and a “typing indicator”**			
		“Just as a general interaction point, when you've asked a question in your text box, it just says ‘Please wait… for a response, but there's no indication that it's actually doing anything, even if it's just like a little animation of thoughts moving”	1	
**Avatar**				
	**Strengths were that the avatar made the chat feel more official or professional**			
		“I think it does make it feel a bit more professional to be fair.”	3	
	**Weaknesses were that to some it felt clinical, weird or unsettling**			
		“A bit medically intense.”	6	
	**Opportunities for development were limited by the program used for development, but suggestions for future work were to have an animal as an avatar, or options for customization**			
		“Maybe there could be an option for turning her off if you just wanted to just have the text.”	5	
		“Do we always have this female? Can we have a male? Can we change the the person who's chatting at us?”	4	
**Speech**				
	**Strengths were that many people found the text-to-speech option helpful**			
		“So I do like that [it] talks to you because I do find that easier rather than having to read through it all.”	2	
	**Weaknesses were that the speech wasn’t helpful for everyone**			
		“It was a little unnatural, a few bits on the intonation”	1	
		“I can't read forward while it's still talking to me sometimes”	3	
	**Opportunities for development were improving the pronunciation, tonality, and being able to mute the speech within the software**			
		“I think if I could click something to just stop it at any point then that'd be huge, because if I was using it for an extended period of time, I'd get quite annoyed with it and I'd probably mute the tab.”	3	
		“Potentially not as like robotic”	6	
**Content**				
	**Strengths were that the chatbot often provided short, punchy messages, several options which used numbering or bullet points, and asked whether the user would like more information**			
		“It keeps it like conversational enough to like, actually listen to it, but it also keeps it quite professional, and it doesn't go into opinions.”	3	
		“That's again really nice, simple piece of advice that sounds great and it's a great little list, so… Positive stuff. Short, concise, nice.”	1	
		“OK, I like it that she's given two options because you can't always be like, here's what you can do. This is what you do. She's actually giving me a moment to sit”	6	
	**Weaknesses were that the information was not always specific enough for example, in terms of regional advice, or the chatbot could not tell users where the information came from**			
		“But actually the practical thing that it doesn't really work that way.”	1	
		“Yeah, this is misleading in terms of real world experience”	1	
		I find it very annoying when somebody gives something as a fact without you being able to find out where that fact came from.”	4	
		“I can look up the NHS^a^ stuff. I can do all that stuff myself. I want to know how to hack the system.”	5	
	**Opportunities for development were being able to trace back information the chatbot gave to its sources, and that it will be important that the chatbot stays up to date with guidance that changes frequently**			
		“a lot of these things on the surface look like they're really helpful, but actually you need quite a lot of scaffolding and support in order to make them work.”	1	
**Chatbox and typing interface**				
	**Strengths were that the chatbot design made it easy to read, and that the chatbot coped fine with typing errors**			
		“I quite like the black with the. Was it green or white or? It was quite easy to read.”	5	
	**Weaknesses were that the chatbot could be glitchy with scrolling, deleting half-written messages, and that the words did not take up much of the screen when compared with the avatar**			
		“She's been a little bit temperamental on whether or not she would move up and down, so I can go back to what we've read before, but she does after a while”	4	
	**Opportunities for development were linking directly to sources of information, making sure it was optimized for phones, and the textbox expanding when writing a long string of text**			
		“Well, I mean, if you had a link straight to the NHS website, you could just click on it. She's not going anywhere. I could click on it. I could look at it and I could come back and ask her a question	4	

^a^NHS: National Health Service.

#### Conversational Flow and Feel

Users liked that the chatbot was able to answer 2-part questions, used numbering in the answers, and that the answers—though lengthy—contained the right amount of information, which appeared to be accurate. Users did not like when the chatbot began replying to their message but then resorted to “Sorry I didn’t quite get that, can you try rephrasing?” halfway through a message. They also didn’t like when the chatbot sent more than one message in a row (though this only happened on one occasion, and appeared to be a technical problem), or when the chatbot restarted and erased the previous history.

To improve the flow of the conversation, users recommended a little animation of thoughts moving while generating a response (collaborator 1), being able to save your chats (collaborator 2), and the chatbot “getting to know” users to personalize responses (collaborator 2).

#### The Avatar

Opinions on the avatar were mixed. Some liked that she looked “official” (Collaborator 4) or “professional” (collaborators 3 and 5). But some people thought that “most people you’re speaking to aren’t going to be in scrubs” (collaborator 4) and found it “distracting” (collaborators 1 and 5) or “unnatural” (collaborator 5) or disliked that the chatbot “wobbled” or had a mouth that moved (collaborator 4). One user found her “not friendly at all” (collaborator 6).

Recommendations for improvement were customization, (collaborators 2 and 4) for example skin color “If you have someone who kind of relates to you, I think it’s easier” (collaborator 2), an option to turn the avatar off and have just the chat, (collaborator 5) having an animal as an avatar, (collaborator 6) or the current one having more expressions (eg, thinking face before answering questions; collaborator 2).

#### The Speech

Users liked that the chatbot speaking aloud was an option. Only one did not use this feature due to the settings with the video call in the think-aloud interview.

However, several users muted the voice after some time interacting. “The only frustration point was not being able to say, “enough talking now” (collaborator 3). People thought the speed of reading was good, but found they could read faster than the chatbot could speak (collaborator 4). Some words, for example, “pediatrician” and abbreviations, for example, “CAMHS” (Child and Adolescent Mental Health Services), and “111” (the United Kingdom phone number for urgent nonemergency medical help) were pronounced incorrectly, and some parts had unnatural intonation.

Users agreed the feature was useful overall, but wanted to add a toggle for pitch (Collaborator 4) and tempo (collaborator 3 and 4). Users also recommended making breaks in the text reflect more clearly in the speech (collaborator 1), the voice being less robotic (Collaborator 6), and with a toggle for switching on or off (Collaborator 3).

#### The Message Content

Feedback was that the content of the messages was positive, with users saying the answers were “short, concise, nice,” “really good,” “comprehensive,” “sensible,” and “useful” (collaborators 1 to 6). Many users liked that the chatbot was concise (collaborator 1 and 6), empathetic (collaborator 5) and used numbering and bullet points to break information down (collaborators 2 and 6). Users expressed skepticism about the chatbot, and did try to ‘test’ it with questions they thought it would not know the answer to. Some users followed up on statements from the chatbot, asking for further details or about information they thought was missing from a response.

Most felt it was pitched correctly and used understandable language (collaborator 6). Users also liked the fact that the chatbot asked questions or provided options for the user about what it could support with next (collaborators 1 and 2).

However, some users felt that not all the information that the chatbot communicated was accurate. This was mostly regarding the provision of health care service information rather than psychoeducation or general information about ADHD. For example, the chatbot was perhaps too optimistic when giving advice: “Not sure that’s necessarily going to be possible” (collaborator 1). Two parents felt that the chatbot should be making people aware that it may not be easy to get support and that you may have to be more “proactive” with your provider (collaborators 4 and 5).

There was also a lack of specificity within some answers about service provision, for example, about regional support. Users suggested this could be overcome with links to useful websites (collaborators 2 and 4), for example, about waiting list lengths, travel to specific countries, or “out of hours” support. Other recommendations for content were to ensure the chatbot mentioned “Right to Choose” (collaborators 1, 3, 4, and 5) as it did not volunteer this information when asked about paths to diagnosis and had to be prompted. Additionally, users acknowledged that some answers were very region-specific.

#### The Chat Box and Typing Interface

Collaborators were positive about the chat box interface, where questions could be typed. The colors, font, and size were readable (collaborators 2 and 6). However, one user did flag that this may be different on phones (collaborator 4). Technical issues included that when users switched between screens, the current message was deleted (collaborator 2). And when typing, this was one continuous bar, so editing questions was difficult (collaborator 3). As the chatbot messages got longer, the chat would jump, which made it difficult to read (collaborator 3).

People recommended the addition of a scroll bar (collaborator 1), allowing files to be attached (collaborator 2), and a zoom function to make the text bigger (collaborator 4).

## Discussion

### Summary of Findings

Initial user feedback on the early prototype of the evidence-based chatbot suggests that this is an avenue that is feasible for future development. Experts by LE (supporters and young people) commented on aspects that they liked, disliked, and future improvements that could be added to the chatbot, particularly about the overall experience, the purpose, the design, the customization settings, and the integration into a future app. Seven key recommendations for future development can be seen in Textbox 2.

**Key recommendations.**

1. An evidence-based chatbot prototype was acceptable and usable for young people living with attention deficit hyperactivity disorder (ADHD) and their supporters, including a supporter who had not used a chatbot before.
2. The chatbot was able to provide psychoeducation, information about accessing health care, and behavioral interventions when requested by users, and users thought that this content was generally good, though information on accessing health care and strategies could be improved.
3. The chatbot was able to tailor advice for users and should be tested further to tailor information to users from underserved groups, or to speak languages that are not English.
4. The ability of the chatbot to offer specific and/or regional advice was limited, for example, waiting times within a local integrated care board for a diagnosis, but this is something that users valued and should be investigated further.
5. Further work will need to concern diversifying the sources of information to provide the chatbot with increased functionality. This could involve the development of an app which acts as a database for evidence-based information, and consultation with health care professionals.
6. Digital poverty and digital literacy need to be addressed in future work so as not to increase health inequalities when designing and proposing the introduction of such technologies more widely.
7. A sustainable funding model is required for development and maintenance of such an innovation.

### Significance

#### Privacy vs Personalization

Collaborators strongly supported the ability of the chatbot to personalize responses to the young person depending on what they input into the chat. However, the ways of doing this whilst maintaining the privacy of the user need further investigation as data protection guidelines produce technical challenges. The research team sees the ability of the chatbot to personalize responses as one of the main benefits of the technology. However, users who are unfamiliar with chatbots or virtual assistants may be unaware of this benefit, and therefore less likely to use it. This could increase health inequalities for individuals who are less technologically literate.

Current studies have also flagged the issue of privacy versus personalization as a key challenge [28,65]. Personalization has been shown to be a key positive feature of other chatbot studies [34]. This was particularly relevant for ethnic minority and non-binary service-users, who are 2 particularly underserved groups [28]. However, no clear solutions are posed in these guidelines. Going forward, using the privacy guidelines of existing successful LLMs could be beneficial for this work. The ANA Chatbot - an AI support tool for ADHD, Autism and AuDHD (Autism and ADHD); for example, already uses the ChatGPT (OpenAI) established data privacy policies and terms, and refers users to these [66].

Conversations about safety more broadly did not occur in the think-aloud interviews, apart from in relation to medication, which contrasts with much of the literature on mental health chatbots, especially around eating disorders. For example, in a 2025 article, Sharp and Dwyer [38] identified safety and risk management as 1 of 4 themes in qualitative interviews when co-designing a chatbot with young people. Potential reasons might include that discussions of ADHD are seen as less emotional or “triggering” than conversations around eating disorders. An additional reason could be that our collaborators in the think-aloud interviews have been involved in the conceptualization of the chatbot and conversations around the limitations and future work required to create a product that was suitable (and safe) for launch.

#### Optimal Functioning of the Chatbot vs Removing Barriers to Access

To improve knowledge of the abilities of the chatbot, information could be provided to users to support optimum or correct use. There is a growing body of literature on how to get the best answers out of AI. The ways of communicating the abilities of the chatbot to users will be key; for example, a user guide to the benefits they can gain from the chatbot. Collaborators felt it was important and useful for instructions to be provided. However, during the think-aloud sessions, users were unsure how instructions should be provided to the user. This contrasted with the importance to people with ADHD of being able to use the chatbot without having to click through menus, messages, or instructions. This also applied to data use policies and setting up a profile so the chatbot has background information on the user. Both would contribute to the optimal functioning of the chatbot but would be barriers to access.

Further consultation is needed on how to tread this line correctly. HCI design principles such as considering “expert users” could be considered when designing these additional features. Other studies also label chatbots as “accessible,” so these could still be better than navigating current systems unaided. Other publications address the issue of instructional guidance for their chatbots [27,31]. Guidelines for designing for ADHD have been published, so these should be considered and, where possible, incorporated in future development [67].

Future work could train LE collaborators in HCI terminology to assist in feedback sessions, and user-testing should be done with multiple versions to identify the most accessible versions.

#### Ability to Customize vs Too Much Choice

The ability to customize features was also a recurring topic of conversation. Throughout the development process, users would make different suggested changes, which we would mock up and present these changes back to users at a later session. When collaborators were then asked to make a choice between the mock-up options, people would often ask, “Why not both of these? They are both good options that would work for different people.” There is further work to be done that explores customization and choice from the perspective of principles of design, and the science of ADHD and choice. To perfect a balance between catering to a wide and diverse audience whilst still maintaining accessibility and ‘usefulness’ will be a key ongoing challenge.

There is some consideration to be given to features that may be accessible to expert users rather than casual users, for example changing the speed of the text-to-speech or the appearance of the avatar. In terms of the conversational aspect, users were happy with the current number of options for next messages. Other publications have explored a blend of structured and open-ended chatbot conversation options [29,68,69].

#### Balancing Evidence-Based, Thorough and Accessible Information

The design of the chatbot was viewed as key for the accessibility of the information. Especially for young people with ADHD, the importance of visual interest and attraction was highlighted by collaborators.

When shown other digital interventions for ADHD, though the information contained was evidence-based, collaborators commented that this would not “work for an ADHD brain.” For example, collaborators suggested that the sentence structures should be different and the level of information was inappropriate (ie, too in-depth), or in some cases even inaccurate. For example, the process of obtaining a diagnosis can be lengthy, taking several years, and is highly variable regionally or by pathway, so the chatbot was unable to offer specific advice. Additionally, some of the information that the chatbot provides may not be relevant to all users and the different support mechanisms they already have in place. This shows the importance of involving experts with LE in the development process, to highlight where NICE guidelines may not reflect the LE. The feedback that information was potentially inaccurate or unrealistic was primarily provided by parents and supporters, who may have more experience navigating the current systems on behalf of the young people they support, and therefore an enhanced ability to identify inaccurate information. It is a crucial point to consider going forward that the potential users of the chatbot will not be experienced in navigating these systems and therefore unable to identify where there is crucial nuance missing from messages. This is especially important as information was flagged by users as “technically correct,” but not necessarily accurate in all cases.

Chatbots can break down tasks into manageable chunks, reducing overwhelm [26,27]. They also offer engagement and motivation [32,33]. This means that they are in a unique position to provide information that is both thorough and nuanced, as well as being engaging and useful.

The combination of nuanced and thorough information with bite-sized messages that are accessible to varied groups will require thorough consideration and user testing in future versions.

#### The Reality of Health Services

While the general psychoeducation information provided by the chatbot was highly approved of by users, some did raise concerns about the extent to which the information about the *health care system* was accurate. This was sometimes because services vary significantly by region, for example the process of getting a diagnosis, waiting list times, and pathways available. However, this was also due to discrepancies between the information provided in NICE guidelines and the realities of how these services are delivered.

In services across the UK, NICE guidelines are “seldom fully implemented,” and services are said to be a “postcode lottery” [70]. Though there are few studies auditing service adherence to guidelines, a previous mapping exercise of adult ADHD services identified that a minority of services offer a comprehensive range of NICE-recommended services [71].

The use of these NICE guidelines as a source of information about health care services should therefore be carefully considered, and alternative sources of information should be appraised in order to provide users with advice that is congruent with the current provision of services.

#### Additional Features of the Chatbot

The authors did not anticipate that users would also ask the chatbot to provide practical tips for managing ADHD (on top of psychoeducation and information about health services). Users in a similar co-design study also thought the chatbot should provide a space to share thoughts and feelings, which was not raised by users in this process, but could feasibly be asked of the chatbot by users. Potts and Ennis [72] suggest that these additional features could be supported by a web app, which could be a future avenue for development of the SmartADHD Chatbot. Similarly, developers of the chatbot “Ebb,” which is associated with the app “Headspace,” emphasize the importance of embedding AI in a larger suite of resources [73].

As Potts and Ennis [72] also highlight, responsible design needs to effectively triangulate the needs of users, what AI is capable of delivering to an acceptable standard, and what mental health professionals will endorse. This is particularly relevant to the provision of behavioral interventions and space to air thoughts and feelings, as these topics can venture into medical advice and therapies. Future development should therefore explore the bounds of what technology is currently capable of delivering to a high standard, the perspectives of HCPs on these abilities, and the ethical implications of offering these other types of support.

### Limitations

The structured nature of the workshops may have influenced the categories chosen and the nature of feedback that was identified. However, this pragmatic approach is appropriate for early intervention development and was necessary to gain specific feedback and actionable suggestions. The think-aloud interviews are likely to have elicited any feedback that did not fit into earlier sessions and, although they followed a brief topic guide, were left as open as possible on purpose.

This was a multidisciplinary project which involved experts by LE, digital experts, HCI experts, clinicians and ADHD researchers. We all come with a wide range of experience and expertise, and though the research team were able to understand the needs and wants of the users, this was sometimes difficult for us to communicate and action due to the mismatch in discipline-specific language between us and the digital team [74]. Further information on challenges and opportunities involved in this type of interdisciplinary working will be discussed in a linked SmartADHD publication.

Versions of the chatbot were developed with inbuilt languages (Arabic, Bengali, Polish, and Romanian) which are commonly spoken as a primary or secondary language in England. Emails were circulated to LE collaborators, colleagues, and a public sector organization, asking for their help in finding a young person who spoke any of these languages fluently (as well as English). However, there were no responses from any of these avenues, so we could not test the functionality of the multilingual chatbot feature. These versions should be tested in the future, especially considering the cultural differences that can apply to ADHD. Differences could include cultural understanding of ADHD, including stigma, parental expectations and social attitudes, differences in help-seeking behavior, and barriers or facilitators to accessing support, though there is a paucity of research in this area [75]. Considering that objective queries were simpler for the chatbot to answer than questions very individual to a user, complex discussions around culture and ADHD will be important to test. Throughout the process of developing the chatbot in English, it was important to people with LE that the chatbot used the right language and semantics. Thus, this work to determine word choice and nuanced meaning would be essential to complete in any alternative languages.

Though there was some diversity across the group in terms of gender, ethnicity, and region, it is critical that any future development work prioritizes inclusion of underserved groups. The potential impacts for these groups are significant, and interventions need to address specific barriers to work for these groups. Additionally, all the users had existing background knowledge of ADHD to varying levels and had been involved in research before. None of the users were completely new to learning about ADHD, so they may represent the intended user group to a limited extent. Additionally, users had been involved with this project since conceptualization and were aware that they were using an early prototype that would require extensive development to be deemed fit for use. Because of this, users were also aware that the options for avatar design using Convai were limited, and so our questions and feedback were concentrated on aspects we could address, for example, language. Due to the restricted choices of the platform, time, and resource constraints, we were unable to fully report on user preferences of the avatar in the role of supporting communication.

This study presents early development of a prototype chatbot, as the main aim was to collect opinion-based feedback to inform co-development, not collect generalizable or transferable data. These opinions will be used to inform the direction of development of digital interventions by this team and may also guide future research in the rapidly evolving area of AI-supported digital mental health interventions for young people [76]. As such, claims about effectiveness cannot be made because the current prototype does not represent a finished product. Future testing of the chatbot could include formalized usability testing, safety assessments, and evaluation of feasibility or performance.

A further limitation is that this initial prototype included less functionality than would be present in the final version. For example, this version was trained only on information from the NHS and the NICE guidelines, whereas future versions could be trained on a more extensive range of evidence sources. Additionally, there could be alternative platforms which could be better suited to hosting the chatbot, both from a developer and a user perspective. This work provides proof of concept for future development, but many questions remain about what the eventual product might look like.

All collaborators who took part in think-aloud interviews were offered the opportunity to provide feedback on the findings by acting as a coauthor on this article, and one accepted. Findings from this engagement research are also being communicated via multiple channels to ensure our LE partners remain informed of developments.

### Conclusions

This study acts as a proof of concept for an LLM chatbot produced specifically to provide support for young people with ADHD. Young people and parent-carers were positive when user-testing a prototype of the chatbot, particularly about the content. Future iterations should aim to state where specific information is from, test its functionality in other languages and with underserved groups in ADHD care, consider improvements to the avatar and text-to-speech function, and address some of the technical glitches. In the space of digital interventions for ADHD, a chatbot could offer a new solution to the existing problem of gaps in health care provision by combining tailored communication and evidence-based information. Key recommendations are presented in Textbox 2.

## Data Availability

Data sharing is not applicable to this article as no data sets were generated or analyzed during this study.

## Appendix

Multimedia Appendix 1 Terms of reference.

Multimedia Appendix 2 Role descriptor.

Multimedia Appendix 3 Logic model.

Multimedia Appendix 4 Intervention planning table.

Multimedia Appendix 5 Chatbot core description.

Multimedia Appendix 6 Chatbot training documents.

Multimedia Appendix 7 Screenshot.

Multimedia Appendix 8 Feedback points.

Multimedia Appendix 9 Chatbot specification document.

## Abbreviations

ADHD: attention deficit hyperactivity disorder
AuDHD: Autism and ADHD
CAMHS: Child and Adolescent Mental Health Services
DHI: digital health intervention
GenAI: generative AI
GP: general practitioner
HCI: human-computer interaction
HCP: health care professional
LE: lived experience
LLM: large language model
MAP: Mapping ADHD services in primary Care
NHS: National Health Service
NICE: National Institute for Health and Care Excellence
NIHR: National Institute for Health and Care Research
PBA: person-based approach
PPIE: patient and public involvement and engagement
RAG: research advisory group
SAND: Science of ADHD and Neurodevelopment
WG: working group
