# Supplementary material for: An Evidence-Based AI Virtual Assistant for Young People With Attention Deficit Hyperactivity Disorder: Co-Design and Prototype Development
Source: JMIR Form Res. 2026 Sep 10;10:e85013. doi: 10.2196/85013 (PMC13613043; doi:10.2196/85013)
Supplement: Supplementary file 1 [file formative_v10i1e85013_app1.docx]

**SmartADHD: Development of a web-app to support young people with ADHD**

**Healthcare professional research advisory group (HP-RAG)**

**Terms of Reference Version 0.1**

1. **Purpose of group**
   a. To help improve healthcare for young people with ADHD
   b. To provide insight, feedback, and support at all stages of the research project
   c. To give RAG members experience of academic research
   d. To help make sure project and findings are shared widely
2. **Membership**
   a. The core group will consist of between 4 and 10 members, who will be invited to attend 10 meetings over 5 years. *There will be a linked working group (WG) (up to 20 members) with opportunities* *to join workshops to help co-develop the SmartADHD resources. RAG members can choose to join WGs.*
   b. Members will be aged 16-30 when joining the RAG, with a diagnosis of ADHD (or awaiting assessment), or be a supporter (e.g. parent/carer) of a young person with ADHD. Members will be recruited from across England to include a diversity of experiences.
   c. Membership will last for the full duration of the research project (approximately 5 years). Members may choose to leave at any time.
3. **Support**
a. The group will be convened by the study lead, Dr Anna Price, and the SmartADHD team.
b. Members will receive an involvement fee for participating, and be offered the opportunity to contribute to study outputs (e.g., app content, publications, plain language summaries, presentations)
c. Members will be offered an introductory meeting that covers the purpose of the research, and the role of the LE-RAG
d. Travel and/or digital access expenses will be paid to enable members to attend face-to-face or virtual meetings
4. **Ways of working**
   a. The LE-RAG will work in accordance with the [NIHR National Standards for Public Involvement](https://sites.google.com/nihr.ac.uk/pi-standards/standards)
   b. RAG meetings will be held online, roughly every 6-months (approximately 10 meetings over 5 years). The research team will communicate with LE-RAG members using a variety of methods to enable ongoing consultation and progress updates, including hosting occasional drop-in sessions.
   c. Research staff will work with lived experience colleagues to set the agenda for each meeting; then take and share notes as quickly as possible (within two weeks).
   d. Research staff will communicate with LE-RAG members so that the group is working with up-to-date information and receives feedback about how its advice has been taken on board
   e. All discussions within the LE-RAG will remain confidential, and details will not be shared unless discussed and agreed within the group

Please note, not everyone who expresses interest will be invited to take part in the LE-RAG. However, you may be offered the opportunity to become a working group (WG) member. Our aim is that this group includes a balance of perspectives (e.g., role, experience, age).

**Glossary**

| **Attention deficit hyperactivity disorder (ADHD)** | Attention deficit hyperactivity disorder (ADHD) is the most common childhood neurodevelopmental disorder. It affects around 5% of children and adolescents, and up to 40% continue to experience symptoms into adulthood. People with ADHD show a persistent pattern of inattention and/or hyperactivity–impulsivity that can interfere with day-to-day functioning and/or development |
| --- | --- |
| **SmartADHD/app** | A project to develop digital resources which provide healthcare information and self-management tools for young people with ADHD |
| **Involvement fee** | A voluntary payment that is given to a person for services for which fees are not legally or traditionally required |
| **Research advisory group (RAG)** | Research advisory groups provide input and advice in the form of thoughts, opinions and ideas to people conducting research about their research projects |
| **LE-RAG** | Lived-experience research advisory group, made up of young people with ADHD, and their supporters (e.g. parents/carers) |
| **Working group (WG)** | Working groups (WGs) provide a structured way for additional input into the SmartADHD project. WG involvement opportunities may arise more frequently than RAG activities, and members are invited to contribute flexibly (depending on their interests and availability). Activities might include, for example, web-app content development/review or a focussed workshop on care pathway planning. RAG members may join study working groups. |
