# Supplementary material for: An Evidence-Based AI Virtual Assistant for Young People With Attention Deficit Hyperactivity Disorder: Co-Design and Prototype Development
Source: JMIR Form Res. 2026 Sep 10;10:e85013. doi: 10.2196/85013 (PMC13613043; doi:10.2196/85013)
Supplement: Supplementary file 2 [file formative_v10i1e85013_app2.docx]

*Lived experience research advisory group (LE-RAG)**. Role Descriptor Version 0.1*

**Are you interested in helping to guide research into development of digital resources for young people (aged 16-25) with ADHD?**

- **Are you a young person (aged 16-30) with ADHD, or do you support a young person with ADHD?**
- **Are you interested in helping researchers develop digital resources for young people with ADHD?**

**What is attention deficit hyperactivity disorder (ADHD)?**

ADHD is a common neurodevelopmental condition that starts during childhood and often persists into adulthood. Symptoms include inattention, hyperactivity, and impulsivity. Experiences of ADHD vary but can include difficulties such as poor organisation, forgetfulness and mood swings. Living with ADHD can interfere with day-to-day living, and people with ADHD are at higher risk of mental health difficulties. Despite highly effective treatments, many young people with ADHD are struggling to access the healthcare and information they need to be able to positively manage their condition.

**What are digital healthcare resources?**

Digital resources can help people to access healthcare and manage their health. The SmartADHD project is developing a web-app that will provide evidence-based healthcare information and self-management tools to improve the quality of life of young people with ADHD.

**What does our research aim to do?**
This project aims to develop a web-app for young people with ADHD (aged 16-25) for use in the NHS. A multi-disciplinary team, including experts by lived experience, will co-develop the app. We will co-create an app that is accessible, inclusive and tailored to the unique needs of individuals with ADHD.

**How can I help?**
We are looking for 4 to 10* young people with ADHD (aged 16-30) and or supporters (parents/carers) of young people with ADHD from across England to be members of the study’s research advisory group. This group will help deliver the project by:

- Shaping research plans and prioritising app content
- Providing feedback on app content, including videos, text, and games
- Helping to share research findings with the community

Group members will be asked to attend 10 meetings during the 5 years of the project. Group members will receive around £25-£35 per meeting, depending on activities. This will cover time for the meeting (about 90 minutes) and activities before or after the meeting.

**We are also looking for working group members, who will be invited to workshops to help co-create content for the web-app.*

**Where do I go for more details or to register an interest?**

- For more information, please visit our website <https://sites.exeter.ac.uk/smartadhd/>
- If you would like to register your interest, please fill in this **short form** by **XXX DATE**
- If you have questions please email the SmartADHD team at [ADHD-research@exeter.ac.uk](https://universityofexeteruk.sharepoint.com/sites/DigitalHealthADHD/Shared%20Documents/05.%20PPIE/4.%20PPI%20documents/ADHD-research@exeter.ac.uk).

Please note, not everyone who expresses interest will be invited to take part in this group. However, you may be offered the opportunity to become a working group member. Our aim is that this group includes a balance of perspectives (e.g., role, experience, age). For opportunities to get involved in linked research, please visit: <https://sites.exeter.ac.uk/adhdresearch/get-involved/>
