# Supplementary material for: An Evidence-Based AI Virtual Assistant for Young People With Attention Deficit Hyperactivity Disorder: Co-Design and Prototype Development
Source: JMIR Form Res. 2026 Sep 10;10:e85013. doi: 10.2196/85013 (PMC13613043; doi:10.2196/85013)
Supplement: Supplementary file 3 [file formative_v10i1e85013_app4.docx]

The SmartADHD App and Chatbot has three target behaviours:

1. Uptake of the SmartADHD App and Chatbot
2. Engaging with SmartADHD Resources
3. Utilising the knowledge/behaviours learnt from resources

**This document encompasses the first two of these goals.**

Sources of Information are colour-coded:

- PPIE Conversations
- Szinay (2020)
- Neurological Characteristics of ADHD
- Consultation with UX

**TDF = Theoretical Domains Network**

| 1. TARGET BEHAVIOUR: Uptake of the SmartADHD App  i.e. downloading the app, opening the app | | |
| --- | --- | --- |
| COM-B Construct   - TDF Elements | **Barriers/*Facilitators*** | **Proposed intervention feature** |
| Physical Capability:   - Skills (physical) | - Motor control* (dexterity to use an app or smartphone) | - User-tested design of buttons etc |
|  | - *Accessibility features for people with co-occurring conditions* | - High contrast, options for different colour palettes. Universal design features. |
| Psychological Capability:   - Knowledge - Skills (cognitive and interpersonal - Memory, Attention, and Decision Processes - Behavioural Regulation | - App Awareness | - Good advertising, referral from Health Care Professional or trusted source. |
|  | - Available User Guidance | - Instructions on how to use the app the first time you open it. |
|  | - Overcoming mental obstacles e.g. stigma | - Positive advertising and positive tone - Using neuro-affirmative language - Positive recommendation from trusted source |
| Physical Opportunity:   - Environmental context and resources | - Cost | - Availability at low cost: Free at point of use |
|  | - *Positive tone* | - Use of language chosen by the LE WG |
|  | - Forgetfulness | - Advertising and a way for people to remember. E.g. a flyer to take away |
|  | - App literacy skills (technological competency to use a smartphone app) | - User tested with a variety of users (Supporters, Healthcare Professionals, Young people with ADHD) |
|  | - Broadband/ signal | - Once downloaded can be used offline |
|  | - Ability to download app or locate on the internet | - Should be able to download on app stores and web version |
| Social Opportunity:   - Social Influences | - Stigma of ADHD (cultural and societal) | - Examples of people with lived experience of ADHD from different backgrounds and different experiences with ADHD |
|  | - Difficult to seek help/support | - Acknowledgement of this being the purpose of the app, gentle encouragement |
|  | - Recommendations | - Referral from Health Care Professional or trusted source |
| Reflective Motivation:   - Beliefs about capabilities - Intentions - Skills - Optimism - Goals - Social/ Professional Role and Identity | - Desire to speak to someone in person / mistrust / distain towards receiving healthcare information from an app | - Positive framing as a clinician-approved resource, a ‘real’ intervention, not just because of lack of other resources |
|  | - *Belief in oneself to engage in a self-led intervention* | - Positive messaging |
|  | - *Caring about the consequences* | - Understanding that app use may make a difference to outcomes |
|  | - *Wanting to make a difference to life/learn* | - Capitalising on this motivation and encouraging this |
| Automatic motivation:   - Reinforcement - Emotions | - *Perceived utility of app* | - Explanation of the mechanisms and why it might be helpful (i.e. increased access to info improves outcomes) |
|  | - *Positive first impression* | - Nice design, helpful starting page – need to consult on what this looks like |
|  | - *Curiosity* | - Some ‘mystery’ in advertising |

| 2. TARGET BEHAVIOUR: Engaging with information about managing ADHD into adulthood, and the UK healthcare system  (future components: Health Tracking, Self-Management, Resource Links, Peer Support Network) | | |
| --- | --- | --- |
| - COM-B Construct - TDF Elements | **Barriers/*Facilitators*** | **Proposed intervention feature** |
| Physical Capability:   - Skills (physical) | - Broadband/Signal | - Once downloaded can be used offline |
| Psychological Capability:   - Knowledge - Skills (cognitive and interpersonal - Memory, Attention, and Decision Processes - Behavioural Regulation | - Temporal Information Processing - Cognitive load | - Clear, concise language to support comprehension - Use of chunked information or multimedia - Features such as progress tracking, reminders and summarisation - Clear, low-clutter interface and intuitive design - Information should be relevant to them – AI chatbot will help |
| Physical Opportunity:   - Environmental context and resources | - Time | - Making the app quick to navigate and easy to find the parts you need |
|  | - Positive Tone | - Neuroaffirmative language and avoiding creating feelings of guilt in users |
|  | - Personalisation | - Features which make it possible for the user to customise the app to their liking |
|  | - Forgetting | - Reminders |
| - Social Opportunity: Social Influences | - Embedded within health professional support | - Provided or prescribed within Primary care systems - Raising awareness in GP surgeries with posters - Promoting nationally and regionally |
|  | - Social networking possibilities | - Online profile, raising awareness of the resource amongst the ADHD community, with - Peer networks and links within the app - Web page - Endorsement by trusted or respected individuals |
| Reflective Motivation:   - Beliefs about capabilities - Intentions - Skills - Optimism - Goals - Social/ Professional Role and Identity | - Motivation regulation | - Reminders with gamification |
|  | - Beliefs about own capability to use a strategy in real life | - Encouragement and stories from LE |
|  | - Intention to seek support | - Covered with stories from LE about when you might want to seek support or engage in a strategy. - Help with planning to seek support? |
|  | - Optimism | - Belief that the resource will be helpful and that change can be made |
| Automatic motivation:   - Reinforcement - Emotions | - Enjoyment of content | - High quality storytelling, good visuals, - Incorporating positive feedback/rewards, see below - Find out from LE what they would enjoy? |
|  | - *Habit-building* | - Reminders (potentially with linked rewards) |
|  | - Guilt | - Gentle reminders |
|  | - Finding content useful | - Content is accurate |
|  | - Trusting the content | - Content is referenced with scientific backing |
|  | - Positive feedback | - Gamification or interactive features e.g. ticking off a section that has been read |
|  | - Available rewards | - Gamification or interactive features e.g. ticking off a section that has been read |
|  | - Goal setting | - Gamification or interactive features e.g. ticking off a section that has been read |
|  | - Perceived utility of app | - Low-clutter interface to reduce cognitive overload - Notifications to build habits - Short-form, quick content |
|  | - Negative emotions towards seeking support | - LE stories of pros and cons of seeking support - LE stories of it working well |
|  | - Fear of failure | - LE stories to cover this - Neuroaffirmative language, it is okay to try and fail |

| **3. TARGET BEHAVIOURS: Utilising the knowledge/behaviours learnt from resources**  **(future components: Health Tracking, Self-Management, Resource Links, Peer Support Network)** | | |
| --- | --- | --- |
| **Physical Capability:** Capacity afforded by the environment (intrinsic) |  |  |
| **Psychological Capability:** The capacity to engage in necessary thought processes – comprehension, reasoning etc. |  |  |
| **Physical Opportunity:** Opportunity afforded by the environment (extrinsic) |  |  |
| **Social Opportunity:**  Opportunity afforded by cultural milieu which dictates the way we think about things |  |  |
| **Reflective Motivation:** Involving evaluations and plans |  |  |
| **Automatic motivation:** Involving emotions and impulses from associative learning and/or innate dispositions |  |  |
