# Supplementary material for: An Evidence-Based AI Virtual Assistant for Young People With Attention Deficit Hyperactivity Disorder: Co-Design and Prototype Development
Source: JMIR Form Res. 2026 Sep 10;10:e85013. doi: 10.2196/85013 (PMC13613043; doi:10.2196/85013)
Supplement: Supplementary file 5 [file formative_v10i1e85013_app8.docx]

**NOTE: This may work better on computers than mobile devices, so ask the user to join on desktop.**

1. Welcome and Introduction
   a. Introduce and welcome
   b. Emphasise you’re testing the chatbot, not the participant
   c. Ask them to verbalise their thoughts as they interact
   d. Encourage both positive and negative feedback
   e. Confirm recording

*“As you use the chatbot, please say out loud what you're thinking—what you're trying to do, what you're expecting, and how you're reacting to what the chatbot says.”*

**Send the user a link to the chatbot. Ask them to open it and share their screen.**

2. Main Task
   a. Let them complete each task while thinking aloud.
   b. Avoid interrupting unless they go silent for a while—then gently prompt:
      i. “What are you thinking right now?”
      ii. “Can you tell me what you expect the chatbot to do next?”
   c. Example of Tasks:

*“Try and get the chatbot to give you information about healthcare for ADHD, for example about…”*

- *how to get support for ADHD from your GP,*
- *how to request a change to your medication,*
- *what to expect from the diagnostic process*

*“Try and get the chatbot to give you advice on managing ADHD, for example about…”*

- *Improving the ability to focus,*
- *Having a hard time with relationships,*
- *or struggling at work*

3. Post-Task Discussion
   a. Ask some prompting questions to get further feedback

**Questions on preferences & usability on the following areas:**

- *Overall interaction, feelings, flow*
- *Content + information*
- *How the chat looks and works*
- *Speech*
- *Video/avatar*

*“What worked well for you?”*

*“Was there anything that didn’t work well for you or was confusing or frustrating?”*

*“Was there anything missing from the chatbot that you would expect to see?”*

*“How would you improve this experience?”*

**Questions on chatbot behaviour and understanding:**

*“Did the chatbot behave the way you expected?”*

*“Did you understand where the chatbot’s information is coming from?”*

a. Let them know what the next steps are & how they will be shaped by their feedback
   i. The next steps are writing this up to share our learning about people’s preferences about the prototype. We will do this in publications in journals
   ii. If you are interested, let me know and we can let you know more about being a co-author on a paper
   iii. The findings from this are going to inform the work that will be going on for the next five years on the SmartADHD app
   iv. There will be opportunities if you would like to present to do this
   v. We are also interested in other ideas for sharing what we have learnt from this project.
b. Let them know that their voucher will be arranged and sent to them by email
c. If they have any further thoughts or feedback, do send us an email
