# Supplementary material for: An Evidence-Based AI Virtual Assistant for Young People With Attention Deficit Hyperactivity Disorder: Co-Design and Prototype Development
Source: JMIR Form Res. 2026 Sep 10;10:e85013. doi: 10.2196/85013 (PMC13613043; doi:10.2196/85013)
Supplement: Supplementary file 6 [file formative_v10i1e85013_app9.docx]

Technical Specification: ADHD Support AI Chatbot

# 1. Purpose and Function

**Primary Function**: Deliver evidence-based psychoeducation, healthcare information, and behavioral support tailored to the ADHD experience.
**Target Users**: Young people with ADHD, newly diagnosed adults, underserved groups (e.g., different cultural, literacy, and socio-economic backgrounds).
**Core Capabilities:**

- Provide ADHD-specific self-management strategies.
- Explain healthcare pathways and the lifelong nature of ADHD
- Support care transitions (e.g., child to adult services).
- Summarise and chunk complex information.
- Personalise content dynamically based on interaction history and user profile.

# 2. Content Architecture

**Evidence Base:**

- All content must be drawn from reliable, clinical, and ADHD-specific resources (e.g., NHS pathways, peer-reviewed psychoeducational material).
- Highlight less commonly known yet evidence-backed information.

**Types of Content:**

- Psychoeducation
- Self-management tools
- Healthcare navigation
- Transition planning
- Lived-experience insights (where appropriate)

# 3. UX Design and Interface

**Tone and Personality:**

- Friendly, non-judgemental, approachable
- Capable of being “fun” if context-appropriate and done well
- Avoid emojis; maintain a professional yet engaging tone
- Clear indication that the bot uses evidence-based information

**Language Style:**

- Unambiguous, plain language
- Technical terms explained before use (e.g., “increasing medication dose (titration)”)
- Sentence limit: ~60–70 characters
- Paragraph limit: ~3 sentences
- Expandable sections for deeper information

**Text Formatting:**

- Important content bolded (in text-color)
- Sans-serif fonts (e.g., Arial, Calibri, Helvetica), bold-weight preferred
- No italics; underlining only for links
- Font size: 12–14pt; inter-letter spacing 0.5–0.9pt

# 4. Visual and Multimedia Components

**Videos:**

- Max 20 seconds, captions mandatory
- Fallback transcript for each

**Images:**

- Alt text required for all visuals
- Use flowcharts and process visuals to summarise complex information

# 5. Personalisation and Adaptability

**Customisation Options:**

- Adjustable content complexity and detail
- Feedback loop (thumbs up/down) to tailor future interactions
- Avatar customisation (e.g., hair, clothes), not overly human-like

Behavioural Tailoring:

- Push relevant content based on life events, age, or symptoms
- Allow discarding certain content types (e.g., motivational, clinical)

# 6. Accessibility and Inclusion

**Designed to cater to diverse needs:**

- Language variants (e.g., English variations, simplified terms)
- most common other languages spoken in England: Polish as first priority, then Bengali, Arabic, and Romanian
- The chatbot shall allow users to specify cultural, regional, or language preferences and adapt content accordingly (e.g., using familiar examples, offering translations, or adjusting for health literacy).
- Options for low literacy or tech literacy users
- Co-occurring conditions considered in both interface and content
- Alternative access formats (e.g., printable summaries)
- Device-adaptive interface that reorganises layout for different screen sizes and supports touch interaction, keyboard navigation, and screen readers

# 7. Functional Capabilities

**Include:**

- Standalone chatbot operation (no app dependency)
- Search function within chatbot history and FAQs
- Max 6 choices per decision-point to avoid overload

# 8. Inclusivity Guidelines for Language

**Use validating language:**

- See table of changes doc for full list: [Table of Chatbot Recommendations for All Workshops.docx](https://universityofexeteruk.sharepoint.com/:w:/r/sites/DigitalHealthADHD/Shared%20Documents/04.%20Research%20projects/02.%20SmartADHD%20Resources/02.%20Chatbot%20development/Table%20of%20Chatbot%20Recommendations%20for%20All%20Workshops.docx?d=w8fe861b2390c44f9be1b0f22e48001d0&csf=1&web=1&e=SlvHGK)
- “Struggles with” > “impairment”
- “Person with ADHD” (with ability to select “ADHDer”, “neurodivergent” in settings)
- Avoid “deficit”, “low functioning”, or “disorder” outside clinical definitions
  Include term glossaries in expandable sections

# 9. Ethical Safeguards and Limitations

**Display clear disclaimers:**

- “This chatbot does not provide medical advice or diagnosis”
- Signpost to live support in case of distress
- Include a privacy page explaining how preferences and interactions are stored
- Include the above as a button that links to a page of ‘FAQs’ or more information
- Data stored locally unless consent given otherwise
- Information should be up to date with the latest NHS/NICE information available

# 10. Future Enhancements

- Integrate reminder or streak functionality carefully (beneficial for engagement but may induce guilt)
- Expand 'remembering’ ability of chatbot – test this with users explore ways of doing this (e.g. profiles)
- Conduct usability testing with underserved and neurodivergent users
- Explore integration into SmartADHD app

# 11. Testing, readability & quality standards

- UX testing requirement. All new features tested with ≥5 users representing different ADHD subtypes, cultural background, and language
